# Kang-Xian Pills Inhibit Inflammatory Response and Decrease Gut Permeability to Treat Carbon Tetrachloride-Induced Chronic Hepatic Injury through Modulating Gut Microbiota

**DOI:** 10.1155/2020/8890182

**Published:** 2020-10-20

**Authors:** Li Wang, Huantian Cui, Yuting Li, Min Cao, Shanshan Man, Liying Guo, Jing Miao, Jianwei Jia, Yuhong Bian, Zhaiyi Zhang

**Affiliations:** ^1^Tianjin Second People's Hospital, Tianjin, China; ^2^Shandong Provincial Key Laboratory of Animal Cell and Developmental Biology, School of Life Sciences, Shandong University, Qingdao, China; ^3^Tianjin University of Traditional Chinese Medicine, Tianjin, China

## Abstract

Kang-Xian (KX) pills have been clinically used for the treatment of chronic hepatic injury (CHI). However, the mechanisms of KX on CHI remain unknown. The aim of this study mainly focused on the anti-inflammatory effects of KX in a CHI mouse model based on modulating gut microbiota and gut permeability. We first established a CHI model using carbon tetrachloride (CCl_4_) and treated it with KX. The anti-inflammatory effects of KX on CHI model mice and the changes in gut permeability after KX treatment were also investigated. 16S rRNA analysis was used to study the changes of gut microbiota composition after KX treatment. In addition, gut microbiota was depleted using a combination of antibiotics in order to further confirm that KX could inhibit the inflammatory response and decrease gut permeability to treat CHI by modulating the gut microbiota. Results showed that KX treatment significantly improved liver function in CHI model mice. KX could also increase the levels of tight junction proteins in the colon and decrease the expression of proinflammatory cytokines in the liver. 16S rRNA analysis indicated that KX treatment affected the alpha and beta diversities in CHI model mice. Further analysis of 16S rRNA sequencing indicated that KX treatment increased the ratio of Firmicutes to Bacteroidetes at the phylum level. At the genus level, KX treatment increased the relative abundance of *Lactobacillus, Bacteroides,* and *Akkermansia* and decreased the relative abundance of *Ralstonia, Alloprevotella,* and *Lachnoclostridium*. However, KX could not alleviate CHI after depleting the gut microbiota. The effects of KX on gut permeability and inflammatory response in the liver were also decreased following the depletion of gut microbiota. In conclusion, our current study demonstrated that gut microbiota was significantly affected during CHI progression. KX could inhibit the inflammatory response and decrease the gut permeability in CHI model mice through modulating the gut microbiota.

## 1. Background

Chronic hepatic injury (CHI) is an inflammatory disease that can be caused by hepatotoxicity, biological factors (hepatitis virus, bacteria, parasite, etc.), chemical factors (medicines, industrial poisons, alcohol, etc.), and environmental factors [1]. Long-term CHI can lead to hepatic fibrosis, liver cirrhosis, hepatocellular carcinoma, and liver failure [2]. Therefore, improving CHI is critical for preventing the occurrence of cirrhosis and liver failure.

Recent studies have shown an important role of gut microbiota dysfunction during the progression of CHI [3]. Carbon tetrachloride- (CCl_4_-) induced CHI was exacerbated in antibiotic-treated mice as compared with mice with conventional gut microbiota [4]. The diversity and relative abundance of gut microbiota also changed in the CHI model [5]. Moreover, some gut microbiota species increased gut permeability and resulted in the release of bacterial metabolites, such as lipopolysaccharide (LPS), into circulation, and the increased level of LPS could contribute to the inflammation in liver [6]. Therefore, modulating gut microbiota is of clinical importance in the study of CHI [6].

Conventional therapies directed against CHI include symptomatic treatment and antibiotics. However, these drugs have side effects that include hepatotoxicity and renal toxicity, and they tend to be less effective in improving liver function [7]. Traditional Chinese medicine (TCM) has been used for the treatment of CHI for thousands of years [8]. Stevia has been shown to be effective in treating CCl_4_-induced CHI by inhibiting oxidative stress [9]. Sheep placental extract inhibits the inflammatory response in a liver injury mouse model [10]. The Taoren-Honghua herbal pair could treat CHI through modulating pathological microvessels and angiogenesis-associated signaling pathways [11]. San-Cao granule showed significant antiapoptotic effects on hepatic injury [12].

Kang-Xian pills (KX), produced by Tianjin Second People's Hospital, contain *Angelica sinensis (*Oliv*.)* Diels*, Ligusticum wallichii* Franch*, Radix Scutellariae, Astragalus membranaceus, Carapax Trionycis, Radix Pseudostellariae, Szechwan Chinaberry Fruit, Schisandra chinensis, Oldenlandia diffusa, Cordyceps sinensis, Paeoniae Rubra Radix,* and *Glycyrrhiza uralensis* and have been used for the treatment of CHI clinically. Our previous study demonstrated that oral treatment of KX (12 g/kg) for four weeks could significantly decrease the serum levels of alanine aminotransferase (ALT) and aspartate aminotransferase (AST) and increase the serum levels of albumin (ALB) and total protein (TP) in CCl_4_-induced CHI model mice. Besides, the infiltration of inflammatory cells and cellular swelling in the liver were significantly improved in KX-treated mice [13]. However, the mechanism of KX action against CHI remains unknown. Considering the important role of gut microbiota in liver inflammation, the aim of this study mainly focused on the anti-inflammatory effects of KX in a CHI mouse model based on modulating gut microbiota and gut permeability. We first established a CHI model using CCl_4_ and treated it with KX. The anti-inflammatory effects of KX on CHI model mice and the changes in gut permeability after KX treatment were also investigated. 16S rRNA analysis was used to study the changes in gut microbiota composition after KX treatment. Additionally, gut microbiota was depleted using a combination of antibiotics to further confirm that KX could inhibit the inflammatory response and decrease gut permeability to treat CHI by modulating the gut microbiota.

## 2. Methods

### 2.1. Reagents

Alanine aminotransferase (ALT), aspartate aminotransferase (AST), albumin (ALB), total protein (TP), and BCA test kits were purchased from Nanjing Jiancheng Biological Engineering Institute (Nanjing, China). Rabbit anti-mouse alpha-smooth muscle actin (*α*-SMA) antibody (ab7817) was purchased from Abcam (Shanghai, China). Rabbit anti-mouse tight junction protein 1 (ZO-1, 61–7300) and occludin (71–1500) antibodies were purchased from Invitrogen (USA). Mouse IL-6, IL-1*β*, and TNF-*α* ELISA kits were obtained from Shanghai BlueGene Biotech Co., Ltd. (Shanghai, China). Total DNA and RNA extraction kits, first-strand cDNA reverse transcription kits, polymerase chain reaction (PCR) kit, and primers were obtained from TianGen Biotechnology Co., Ltd. (Beijing, China).

### 2.2. Preparation of KX

**Figure 1:**
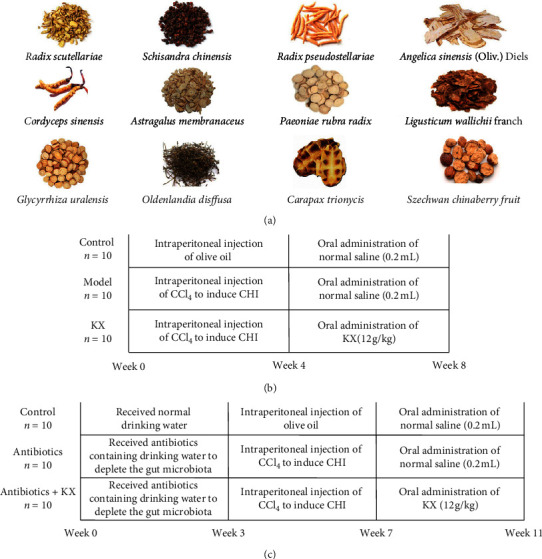
Overview of the experimental design for all groups: (a) drugs in KX; (b) mice received CCl_4_ injection to induce a CHI model followed by oral KX treatment; (c) mice first received a combination of antibiotics diluted in drinking water to deplete the gut microbiota. Then, mice received CCl_4_ injection to induce a CHI model followed by oral KX treatment.

KX pills were prepared from the pharmacy department of Tianjin Second People's Hospital. Briefly, *Angelica sinensis (*Oliv*.)* Diels (Tianjin traditional Chinese Medicine prepared pieces Co., Ltd., Tianjin, China, Batch number: 1901026), *Ligusticum wallichii* Franch (Tianjin traditional Chinese Medicine prepared pieces Co., Ltd., Tianjin, China, Batch number: 1812026), *Radix Scutellariae* (Tianjin traditional Chinese Medicine prepared pieces Co., Ltd., Tianjin, China, Batch number: 1901022), *Astragalus membranaceus* (Tianjin traditional Chinese Medicine prepared pieces Co., Ltd., Tianjin, China, Batch number: 1902021), *Carapax Trionycis* (Tianjin traditional Chinese Medicine prepared pieces Co., Ltd., Tianjin, China, Batch number: 1901012), *Radix Pseudostellariae* (Tianjin traditional Chinese Medicine prepared pieces Co., Ltd., Tianjin, China, Batch number: 1812005), *Szechwan Chinaberry Fruit* (Tianjin traditional Chinese Medicine prepared pieces Co., Ltd., Tianjin, China, Batch number: 1901023), *Schisandra chinensis* (Tianjin traditional Chinese Medicine prepared pieces Co., Ltd., Tianjin, China, Batch number: 1901024), *Oldenlandia diffusa* (Tianjin traditional Chinese Medicine prepared pieces Co., Ltd., Tianjin, China, Batch number: 1812016), *Cordyceps sinensis* (Tianjin traditional Chinese Medicine prepared pieces Co., Ltd., Tianjin, China, Batch number: 1812001), *Paeoniae Rubra Radix* (Tianjin traditional Chinese Medicine prepared pieces Co., Ltd., Tianjin, China, Batch number: 1812005), and *Glycyrrhiza uralensis* (Tianjin traditional Chinese Medicine prepared pieces Co., Ltd., Tianjin, China, Batch number: 1901026) were weighed according to the medical institution preparation standard in Tianjin (approval number: Z20070138). All herbs were authenticated by Pharmacist Li Wang in the Department of Pharmacy of the Tianjin Second People's Hospital. Then, herbs were crushed and sterilized for 12 h to obtain KX (Figure 1(a)).

### 2.3. Animals

Male C57BL/6 mice, weighing 20.0 ± 2.0 g, were obtained from Beijing Huafukang Animal Company. All animals were handled in accordance with the experimental protocols outlined by the National Institutes of Health regulation and approved by the Ethics Committee and Use Committee of the Tianjin University of Traditional Chinese Medicine.

### 2.4. Induction of CHI Using CCl_4_

After 3 days of acclimatization, CHI was induced as described previously [13]. In brief, the mice received intraperitoneal injections of 20% CCl_4_ solution diluted in olive oil (2 mL/kg body weight), twice weekly for a four-week period.

### 2.5. Depletion of the Gut Microbiota

The gut microbiota was depleted using a combination of antibiotic treatments as described previously [14]. Briefly, mice were given a combination of ciprofloxacin (0.2 g/L) and metronidazole (1 g/L) for 3 weeks in their drinking water.

### 2.6. Animal Grouping

Mice were randomly divided into three groups (*n* = 10 per group): Control, Model, and KX groups. The Model and KX groups received CCl_4_ injections to induce CHI. After four weeks of CCl_4_ injections, mice in the Model and KX groups received treatment orally of either 0.2 mL normal saline or KX (12 g/kg) once per day for four weeks, respectively. The Control group received an intraperitoneal injection of 0.2 mL olive oil twice per week for four weeks followed by oral treatment of 0.2 mL normal saline once per day for four weeks (Figure 1(b)).

For the gut microbiota depletion experiment, mice were randomly divided into three groups (*n* = 10 per group): Control, Antibiotics, and Antibiotics + KX. Mice in the Antibiotics and Antibiotics + KX groups were treated with a combination of antibiotics to deplete their gut microbiota followed by intraperitoneal injection of CCl_4_ to induce CHI. After four weeks of CCl_4_ injections, mice in the Antibiotics and Antibiotics + KX groups received treatment orally of either 0.2 mL normal saline or KX (12 g/kg) once per day for four weeks, respectively. Mice in the Control group remained untreated for three weeks followed by intraperitoneal injected of 0.2 mL olive oil twice per week for four weeks. After four weeks of olive oil injection, mice in the Control group received treatment orally of 0.2 mL normal saline once per day for four weeks (Figure 1(c)).

At the end of the four weeks of KX treatment, mice were anesthetized with ether and blood was obtained from the retrobulbar plexus for serum analysis. Then, mice were sacrificed under anesthesia, and livers and colon tissues were obtained for pathological and molecular biological studies. The liver index was calculated using the following formula: liver index (%) = liver weight (g)/body weight (g) × 100.

### 2.7. Serum Biochemical Analysis

Blood samples were centrifuged at 3,000 rpm for 15 min to obtain serum. The levels of ALT, AST, ALB, and TP in serum were measured by commercial test kits according to the manufacturer's protocol [13]. The absorbance value was determined using a microplate reader.

### 2.8. Histology

Mouse livers were fixed in paraformaldehyde after KX treatment. The liver was embedded in paraffin and subsequently cut into 5 *μ*m sections. Sections were stained with hematoxylin and eosin (H&E) and Masson. The ratio of collagenous fiber area to sum area in Masson staining was analyzed and quantified using ImageJ based on the integrated optical density (IOD).

### 2.9. Immunostaining

The expression of alpha-smooth muscle actin (*α*-SMA) in the liver and the expression of occludin and tight junction protein-1 (ZO-1) in the colon were accessed using immunostaining. The ratio of positive expressed area to sum area was analyzed and quantified using ImageJ based on the IOD.

### 2.10. Cytokine Quantification by Enzyme-Linked Immunosorbent Assay (ELISA)

Liver tissue (0.1 g) was weighed and put into 900 mL normal saline followed by ultrasonic trituration and centrifugation at 3,000 rpm for 15 min to obtain tissue homogenate. The level of the total protein content in liver homogenate was detected by BCA assay according to the manufacturer's instructions (Nanjing Jiancheng Bioengineering Institute). The levels of IL-1*β*, IL-6, and TNF-*α* in the tissue homogenate were measured using ELISA according to the manufacturer's instructions (Shanghai BlueGene Biotech Co., Ltd. China). Cytokine concentrations in liver homogenates were expressed as relative values to the total protein concentration [15, 16].

### 2.11. RNA Isolation and Real-Time Reverse Transcription Quantitative Polymerase Chain Reaction (qPCR)

According to the manufacturer's instructions, total RNAs were isolated from the mouse livers using an RNA extraction kit. The first-strand cDNA was synthesized using 1 *μ*g of total RNA. Real-time reverse transcription-quantitative polymerase chain reaction (qPCR) was used to measure the expression of *IL-1β*, *IL-6,* and *TNF-α* in the liver as previously described [14]. All samples were performed in triplicate and detected using a BioRad iQ5 Detection System. *β-actin* was used as a loading control. Quantification was performed using the 2^−△△CT^ method [17]. The sequences of the primers were listed in Table 1.

### 2.12. Fecal 16S rRNA Sequencing

After KX treatment, feces from the Control, Model, and KX groups were simultaneously obtained under sterile conditions in a laminar flow hood. Total DNAs were extracted from fecal samples and the 16S rRNA sequencing was conducted as described previously [18].

### 2.13. Statistics

All data were reported as the mean ± standard deviation (mean ± SD) for independent experiments. Statistical differences between the experimental groups were examined by analysis of variance (ANOVA) using SPSS version 20.0 (SPSS, Inc., Chicago, IL, USA). A *p* value <0.05 was considered statistically significant. Curve fitting was carried out using the graphical package GraphPad Prism5 (GraphPad Software, Inc., La Jolla, USA).

## 3. Results

### 3.1. Effect of KX on CHI Mice

**Figure 2:**
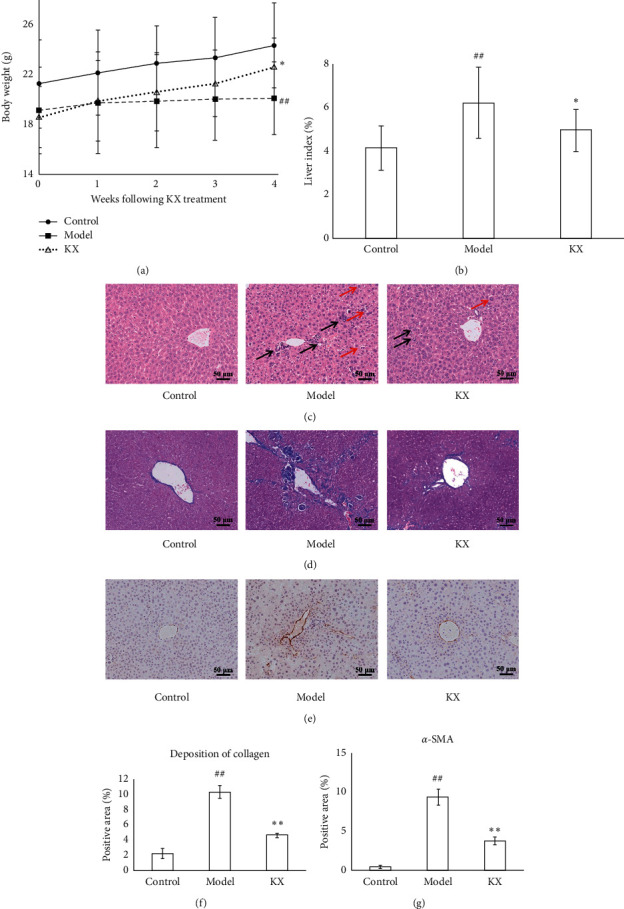
KX treatment improved the pathological changes in CHI model mice. (a, b) KX treatment increased the body weight and liver index CHI model mice. (c) H&E staining indicated that KX treatment ameliorated the inflammatory cells infiltration and cellular swelling of hepatocytes. Black arrows indicate the inflammatory cell infiltration. Red arrows indicate the cellular swelling of hepatocytes. (d, f) Masson staining indicated that KX treatment decreased the collagen deposition in the liver. (e, g) Immunostaining indicated that KX treatment decreased the levels of *α*-SMA in the liver (100x). Control, Model, and KX groups (*n* = 10 per group). Data are presented as mean ± SD. ^##^*p* < 0.01 compared with the Control group; ^*∗*^*p* < 0.05 compared with the Model group; ^*∗∗*^*p* < 0.01 compared with the Model group.

After four weeks of KX treatment, serum levels of AST, ALT, ALB, and TP were investigated to assess the therapeutic effects of KX on CHI mice. Results showed that the body weight in the Model group was lower than that in the Control group (*p* < 0.01), whereas the body weight was increased in CHI model mice after 4 weeks of KX treatment (*p* < 0.05, Figure 2(a)). The liver index was higher in the Model group compared with the Control group (*p* < 0.01, Figure 2(b)). KX treatment decreased the liver index in CHI model mice (*p* < 0.05, Figure 2(b)). Additionally, the serum levels of AST and ALT were increased (*p* < 0.01, Table 2), and the serum levels of ALB and TP were decreased (*p* < 0.05 and *p* < 0.01, Table 2) in the Model group as compared with the Control group. KX treatment decreased the serum levels of AST and ALT (*p* < 0.01, Table 2) and increased the serum levels of ALB and TP (*p* < 0.05, Table 2) in CHI model mice. Mouse livers were stained with H&E to observe the pathological changes after KX treatment. H&E staining showed obvious infiltration of inflammatory cells and cellular swelling of hepatocytes in the Model group, whereas KX treatment ameliorated the inflammatory cell infiltration and cellular swelling of hepatocytes (Figure 2(c)). Masson staining showed an obvious deposition of collagen in the liver of CHI model mice, whereas the deposition of collagen in the liver was significantly reduced in KX-treated mice as compared to the CHI model mice (*p* < 0.01, Figures 2(d) and 2(f)). Immunostaining also showed that the expression of *α*-SMA in the liver was increased more in the Model group than that in the Control group (*p* < 0.01, Figures 2(e) and 2(g)), KX treatment significantly decreased the expression of *α*-SMA in the liver compared with the Model group (*p* < 0.01, Figures 2(e) and 2(g)).

### 3.2. Effects of KX on Gut Permeability and Levels of Proinflammatory Cytokines in the Liver

**Figure 3:**
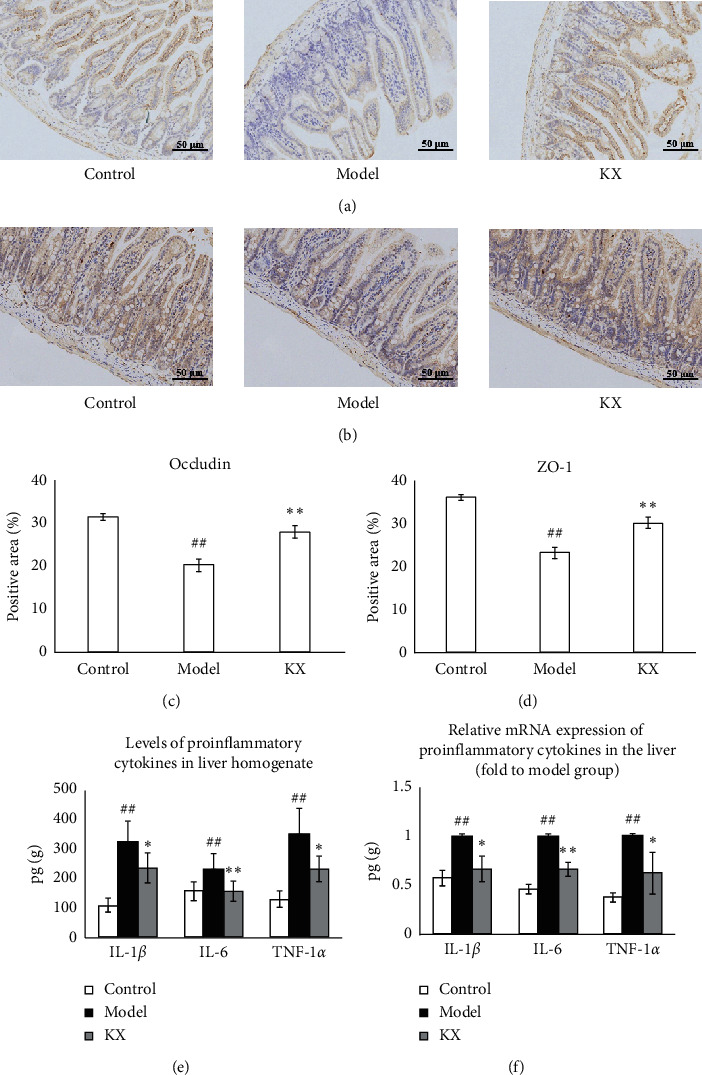
KX treatment increased gut permeability and inhibited the levels of proinflammatory cytokines in CHI model mice. Immunostaining indicated that KX treatment increased the levels of occludin (a, c) and ZO-1 (b, d) in the colon (200x). (e, f) KX treatment decreased the levels of IL-1*β*, IL-6, and TNF-*α* in liver homogenate and downregulated the gene expression of *IL-1β, IL-6,* and *TNF-α* in the liver. Control, Model, and KX groups (*n* = 10 per group). Data are presented as mean ± SD. ^##^: *p* < 0.01 compared with the Control group; ^*∗*^*p* < 0.05 compared with the Model group; ^*∗∗*^*p* < 0.01 compared with the Model group.

The effects of KX on gut permeability were investigated using immunostaining. Results showed that the levels of occludin (*p* < 0.01, Figures 3(a) and 3(c)) and ZO-1 (*p* < 0.01, Figures 3(b) and 3(d)) in the colon were decreased in the Model group compared with the Control group, and KX treatment increased the levels of occludin (*p* < 0.01, Figures 3(a) and 3(c)) and ZO-1 (*p* < 0.01, Figures 3(b) and 3(d)) in the colon. In addition, the levels and mRNA expression of IL-1*β*, IL-6, and TNF-*α* in the liver were measured to observe the anti-inflammatory effects of KX on CHI. The levels of IL-1*β*, IL-6, and TNF-*α* were significantly increased in the Model group compared with the Control group (*p* < 0.01, Figure 3(e)), and KX treatment decreased the levels of IL-1*β*, IL-6, and TNF-*α* in liver homogenate (*p* < 0.05, *p* < 0.01, *p* < 0.01, respectively, Figure 3(e)). Likewise, the mRNA expression of *IL-1β*, *IL-6,* and *TNF-α* was higher in the Model group (*p* < 0.01, Figure 3(f)), and KX treatment downregulated the gene expression of *IL-1β*, *IL-6,* and *TNF-α* in the liver (*p* < 0.05, *p* < 0.01, *p* < 0.05, respectively, Figure 3(f)).

### 3.3. KX Treatment Influenced the Gut Microbiota Community in CHI Mice

**Figure 4:**
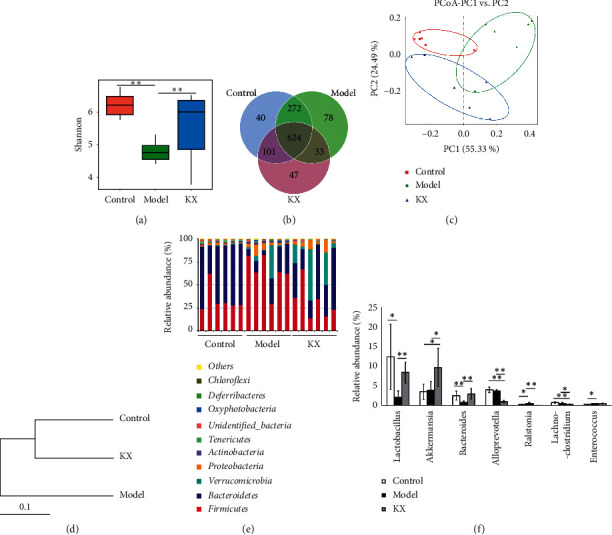
KX treatment changed the diversity and abundance of gut microbiota in the CHI mouse model: (a) the Shannon index was lower in the Model group as compared to the Control and KX groups; (b) the Venn diagram indicates the differential numbers of OTUs in each group; (c) PCoA score based on weighted UniFrac metrics was different in each group; (d) system clustering tree of gut microbiota based on weighted UniFrac metrics indicates the different beta diversity of gut microbiota in each group; (e) KX treatment changed the microbial community at the phylum level (bar plot); (f) at the genus level, KX treatment increased the relative abundances of *Lactobacillus*, *Bacteroides,* and *Akkermansia* and decreased the relative abundances of *Ralstonia, Alloprevotella,* and *Lachnoclostridium* in the CHI mouse model, the relative abundance of *Enterococcus* increased in the Model group, and KX treatment did not affect the relative abundance of *Enterococcus* in the CHI mouse model. Control, Model, and KX groups (*n* = 6 per group). Data are presented as mean ± SD. ^*∗*^*p* < 0.05; ^*∗∗*^*p* < 0.01.

High-throughput sequencing of 16S rRNA was conducted to study the changes in gut microbiota in CHI model mice after KX treatment. We obtained 1,383,228 usable reads and 1,195 operational taxonomic units (OTUs) from 18 samples. The Shannon diversity index was higher in the Control and KX groups than that in the Model group (Figure 4(a)). Moreover, the Venn diagram revealed that 624 OTUs were common to all three groups, 896 OTUs were present in both the Control and Model groups, 725 OTUs were present in both the Control and KX groups, and 657 OTUs were present in both the Model and KX groups (Figure 4(b)). Principal coordinates analysis (PCoA) showed a significant difference in gut microbiota in each group (Figure 4(c)). System clustering trees showed that the distance between the Control and KX groups was closer than that between the Control and Model groups (Figure 4(d)).

We further investigated the relative abundance of gut microbiota. At the phylum level, 10 phyla were found in all samples, and the most abundant phyla in all samples were Bacteroidetes and Firmicutes (Figure 4(e)). The Firmicutes to Bacteroidetes ratio (*F* to *B* ratio) was significantly higher in the Model group than that in the Control group. KX treatment significantly decreased the *F* to *B* ratio as compared with the Model group (Figure 4(e)). At the genus level, the relative abundances of *Lactobacillus* and *Bacteroides* were lower (*p* < 0.05 and *p* < 0.01, respectively, Figure 4(f)) and the relative abundances of *Enterococcus* and *Ralstonia* were higher (*p* < 0.05, Figure 4(f)) in the Model group than those in the Control group. However, the KX treatment increased the relative abundances of *Lactobacillus* and *Bacteroides* (*p* < 0.01, Figure 4(f)) and decreased the relative abundances of *Ralstonia* (*p* < 0.01, Figure 4(f)) in CHI model mice. In addition, KX treatment remarkably increased the relative abundance of *Akkermansia* (*p* < 0.05, Figure 4(f)) and decreased the relative abundance of *Alloprevotella* (*p* < 0.01, Figure 4(f)) and *Lachnoclostridium* (*p* < 0.05 and *p* < 0.01, respectively, Figure 4(f)) compared with both the Model and Control groups.

### 3.4. Effects of KX on CHI following the Depletion of Gut Microbiota

**Figure 5:**
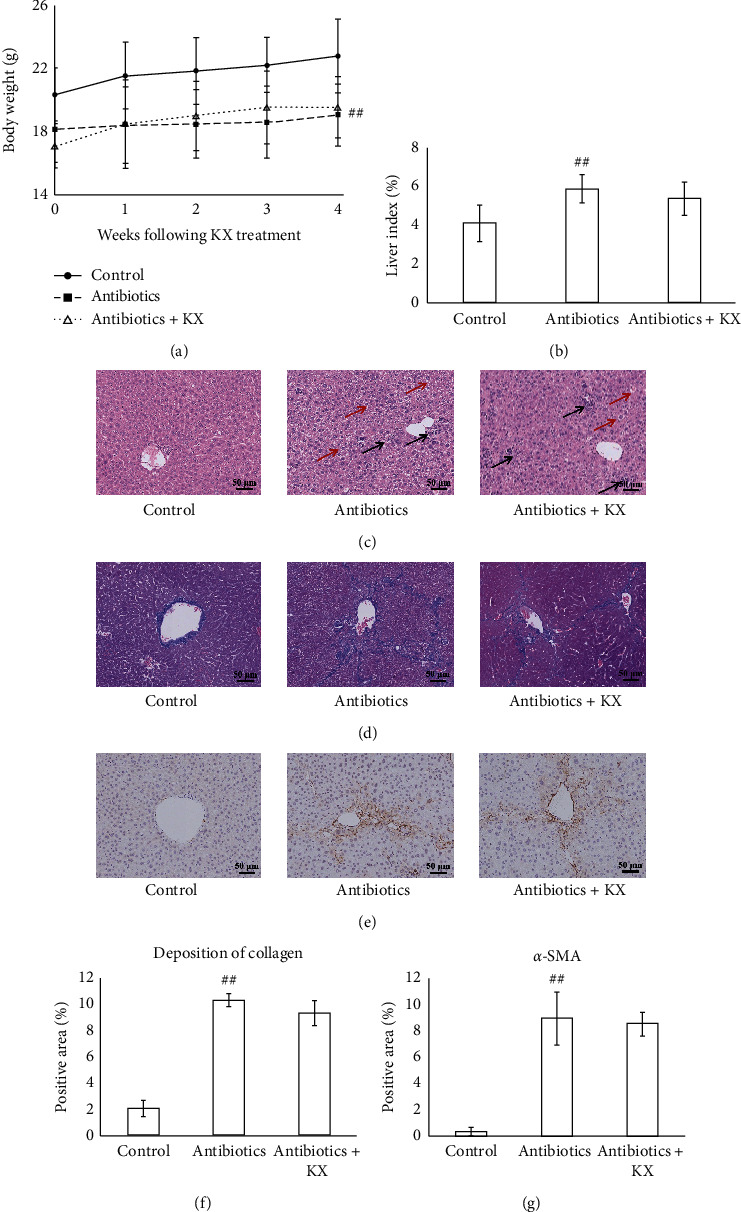
KX treatment did not affect the pathological changes after the depletion of gut microbiota. (a, b) KX treatment did not affect the body weight and liver index in CHI model mice after depleting the gut microbiota. (c) H&E staining indicated that KX treatment did not affect the inflammatory cell infiltration and cellular swelling of hepatocytes after depleting the gut microbiota. Black arrows indicate the inflammatory cell infiltration. Red arrows indicate the cellular swelling of hepatocytes. (d, f) Masson staining indicated that KX treatment did not affect the deposition of collagen in the liver after depleting the gut microbiota. (e, g) Immunostaining indicated that KX treatment did not affect the level of *α*-SMA in the liver after depleting the gut microbiota (100x). Control, Antibiotics, and Antibiotics + KX groups (*n* = 10 per group). Data are presented as mean ± SD. ^##^*p* < 0.01 compared with the Control group.

A combination of antibiotics was used to deplete the gut microbiota followed by CCl_4_ and KX treatment to further validate KX's role in improving CHI through modulating gut microbiota. The results showed that the body weight was lower (*p* < 0.01, Figure 5(a)) and the liver index (*p* < 0.05, Figure 5(b)) was higher in the Antibiotics group than those in the Control group. The serum levels of AST and ALT were increased (*p* < 0.01, Table 3) and the serum levels of ALB and TP were decreased (*p* < 0.05, Table 3) in the Antibiotics group as compared with the Control group. In addition, following the depletion of gut microbiota, KX treatment did not affect the body weight (not statistically significant, Figure 5(a)), liver index (not statistically significant, Figure 5(b)), and serum levels of AST, ALT, ALB, or TP (not statistically significant, Table 3) in CHI model mice. H&E staining showed obvious infiltration of inflammatory cells and cellular swelling of hepatocytes in the Antibiotics group, whereas there were no significant differences in inflammatory cell infiltration and cellular swelling of hepatocytes between the Antibiotics and Antibiotics + KX groups (Figure 5(c)). In addition, the Masson staining showed an obvious deposition of collagen in the Antibiotics group (*p* < 0.01, Figures 5(d) and 5(f)), whereas there were no significant differences in collagen deposition between the Antibiotics and Antibiotics + KX groups (not statistically significant, Figures 5(d) and 5(f)). Immunostaining also showed that the expression of *α*-SMA in the liver was increased in the Antibiotics group than that in the Control group (*p* < 0.01, Figures 5(e) and 5(g)). There were no significant differences in *α*-SMA expression between the Antibiotics and Antibiotics + KX groups (not statistically significant, Figures 5(e) and 5(g)).

### 3.5. Effects of KX on Gut Permeability and the Levels of Proinflammatory Cytokines in the Liver following the Depletion of Gut Microbiota

**Figure 6:**
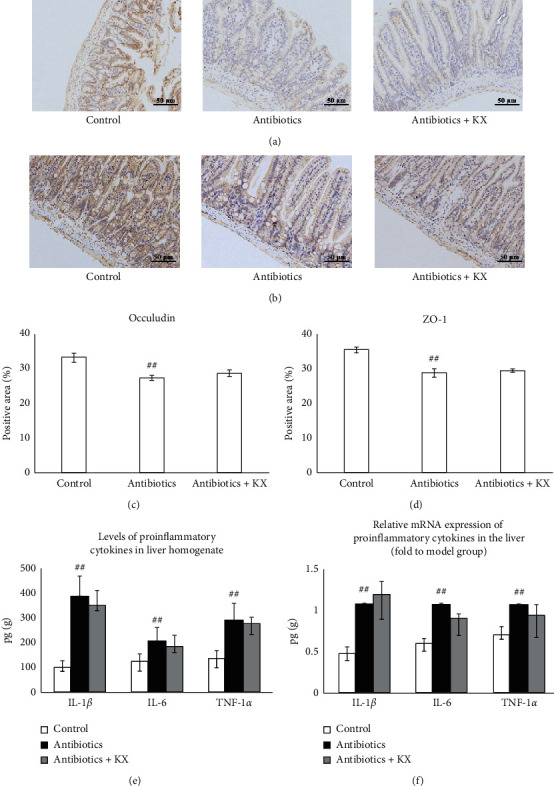
KX treatment did not affect the gut permeability and the levels of proinflammatory cytokines after depletion of gut microbiota. Immunostaining indicated that KX treatment did not affect the levels of occludin (a, c) and ZO-1 (b, d) in the colon after depletion of gut microbiota (200×). (e, f) KX treatment did not affect the levels of IL-1*β*, IL-6, and TNF-*α* in liver homogenate and the gene expression of *IL-1β, IL-6,* and *TNF-α* in the liver after depletion of gut microbiota. Control, Antibiotics, and Antibiotics + KX groups (*n* = 6 per group). Data are presented as mean ± SD. ^##^*p* < 0.01 compared with the Control group.

Results showed that the levels of occludin (*p* < 0.01, Figures 6(a) and 6(c)) and ZO-1 (*p* < 0.01, Figures 6(a) and 6(d)) in the colon were decreased in the Antibiotics group compared with the Control group. There were no significant differences in the levels of occludin (not statistically significant, Figures 6(a) and 6(c)) and ZO-1 (not statistically significant, Figure 6(a) and 6(d)) between the Antibiotics and Antibiotics + KX groups. In addition, the levels of IL-1*β*, IL-6, and TNF-*α* were significantly increased in the Antibiotics group compared with the Control group (*p* < 0.01, respectively, Figure 6(e)). There were no significant differences in the levels of IL-1*β*, IL-6, and TNF-*α* between the Antibiotics and Antibiotics + KX groups (not statistically significant, Figure 6(e)). The mRNA expression of *IL-1β*, *IL-6,* and *TNF-α* was higher in the Antibiotics group (*p* < 0.01, respectively, Figure 6(f)). There were no significant differences in the gene expression of *IL-1β*, *IL-6,* and *TNF-α* between the Antibiotics and Antibiotics + KX groups (not statistically significant, Figure 6(f)).

## 4. Discussion

In this study, we established an animal model of CHI using CCl_4_ injection. Our results showed that the mice in the Model group exhibited abnormal biomarkers in serum, characterized by an increase of AST and ALT and a decrease of ALB and TP. The body weight was decreased in CHI model mice. Our results also showed a significant infiltration of inflammatory cells, cellular swelling of hepatocytes, and collagen deposition in the Model group. The level of *α*-SMA was also increased in the Model group. *α*-SMA is highly expressed in fibrocytes. Increased level of *α*-SMA has been demonstrated to be associated with the progression of liver fibrosis and liver cirrhosis. These results were in agreement with the clinical features and pathological changes in CHI [19]. Consistent with our previous studies, the KX treatment alleviated the abnormal serum biomarkers and pathological changes and decreased the levels of *α*-SMA in CHI model mice.

Studies have shown that CCL_4_ could induce the release of proinflammatory cytokines such as IL-1*β*, IL-6, and TNF-*α* in the liver and further activate quiescent hepatic stellate cells (HSCs) and Kupffer cells [20]. The activated HSCs and Kupffer cells could in turn promote inflammatory and fibrogenic responses [21]. Decreasing the levels of IL-1*β*, IL-6, and TNF-*α* could alleviate CHI and hepatic fibrosis [15, 22]. Likewise, higher expression of proinflammatory cytokines (IL-1*β*, IL-6, and TNF-*α*) was observed in CHI model mice and KX treatment decreased the expression of proinflammatory cytokines in the liver. The dysfunction of gut microbiota could impair the permeability of the intestinal epithelial cell barrier and contribute to the inflammatory response in the liver [6]. Decreasing the gut permeability could alleviate the inflammatory response in the liver [23]. Our results showed that the gut permeability was increased in CHI model mice and KX could decrease the gut permeability through increasing the levels of ZO-1 and occludin in the colon. ZO-1 and occludin are tight junction proteins expressed in the intestinal epithelial cells [24]. Occludin could control the intestinal epithelial tight junction barrier through regulating macromolecule flux [25]. ZO-1, also called tight junction protein 1, is a cytoplasmic plaque protein and connects the transmembrane proteins to the cytoskeleton [26, 27].

In addition, we investigated the changes in microbiological composition using high-throughput sequencing. Our results demonstrated that the alpha diversity of the gut microbiota community was decreased in the Model group, which had a lower Shannon Diversity index than the Control group. The Shannon diversity index in the KX group was higher than the Model group, which indicated that the KX treatment increased the alpha diversity in the CHI mouse model. PCoA analysis revealed significant distances between all groups, indicating that the beta diversity of gut microbiota of the CHI mouse model and KX-treated mice differed from that of the Control mice. According to the system clustering tree, the beta diversity between the Control and KX groups was similar to that between the Model and Control groups. Our results also showed that the *F* to *B* ratio was increased in the CHI mouse model and was decreased after KX treatment. It has been demonstrated that the ratio of *F* to *B* was increased in nonalcoholic fatty liver diseases (NAFLD) [28]. However, the correlations of the *F* to *B* ratio in CHI require further study. We also found that the relative abundances of *Lactobacillus*, *Bacteroides*, and *Akkermansia* were increased after KX treatment. *Lactobacillus* is a type of probiotic and has been used to treat many liver diseases including CHI [29], NAFLD [30], alcoholic liver disease [31], and acute liver injury [32]. Oral treatment of *Lactobacillus rhamnosus GG* could ameliorate liver fibrosis and inflammatory response in rats [33]. The anti-inflammatory effect of KX on CHI might be through increasing the abundance of *Lactobacillus* in the gut. *Bacteroides* has been shown to produce many metabolites, such as short-chain fatty acids (SCFAs) [34] and polysaccharide A (PSA) [35]. Studies have shown that *Bacteroides fragilis* could protect against *Bartonella henselae*-induced liver damage through producing PSA [36]. SCFAs are derived from indigestible carbohydrates by the fermentation of gut microbiota and include compounds such as butyrate, propionate, and acetate [37]. Accumulated studies have reported the anti-inflammatory effects of SCFAs. Butyrate could relieve colonic inflammation through inducing T regulatory cell (Treg) differentiation [38]. Additionally, SCFAs could suppress cholesterol synthesis in rat liver [39]. In contrast, studies also show that the relative abundance of *Bacteroides* was increased in nonalcoholic steatohepatitis (NASH) and at the end stage of hepatic fibrosis in patients [40]. Therefore, the detailed role of *Bacteroides* in CHI should be investigated in future studies. *Akkermansia* has been shown to increase the integrity of the intestine epithelial layer [41]. Moreover, *Akkermansia muciniphila* could prevent obesity and its associated metabolic disorders [42]. Recent studies demonstrated that *Akkermansia muciniphila*-derived extracellular vesicles could enhance the tight junction function on diabetic model mice. *In vitro* studies also showed that *Akkermansia muciniphila*-derived extracellular vesicles could decrease the gut permeability on human intestinal epithelial cells [43]. Increasing the abundance of *Akkermansia* might be the mechanism of KX on gut permeability. Our results also indicated that the relative abundance of *Enterococcus* was increased in the CHI mouse model. *Enterococcus* has been shown to cause empyema in a patient with liver disease [44]. Proton pump inhibitors could promote liver injury by inducing the overgrowth of *Enterococcus* [45]. Moreover, KX treatment decreased the relative abundance of *Ralstonia*, *Alloprevotella*, and *Lachnoclostridium*. Few studies have shown the relationship between *Ralstonia, Alloprevotella,* and *Lachnoclostridium* in CHI, which requires further studies. These results indicated that KX could affect the gut microbiota, decrease the gut permeability, and inhibit the inflammatory response in CHI model mice.

To further verify that KX could decrease the gut permeability and inhibit the inflammatory response to treat CHI through affecting gut microbiota, we used antibiotics to deplete the gut microbiota. Our previous studies demonstrated that a combination treatment of antibiotics could deplete most of the gut microbiota in mice [14], and this method did not influence the histological profiles of the liver, kidney, small intestine, or colon in mice [14]. Our current results showed that KX had a decreased effect on CHI following the depletion of gut microbiota, indicating that KX treatment did not alleviate CHI after the depletion of gut microbiota. Consistently, the effects of KX on gut permeability and inflammatory response in the liver were also decreased following the depletion of gut microbiota.

**Figure 7:**
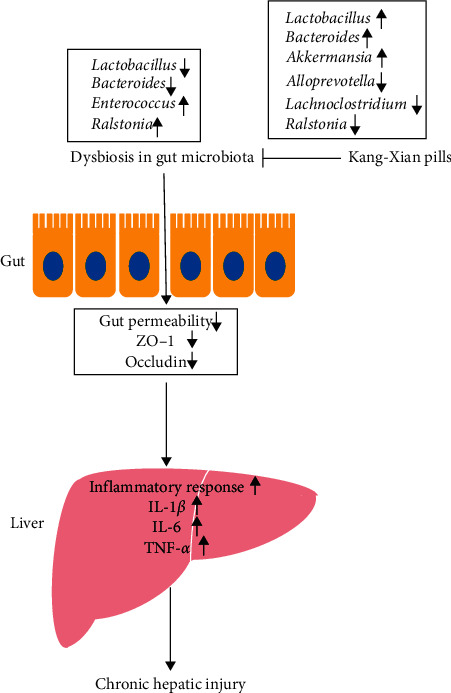
Graphical abstract.

In conclusion, our current study demonstrated that gut microbiota was significantly affected during CHI progression. KX could inhibit the inflammatory response and decrease the gut permeability in CHI model mice through modulating the gut microbiota (Figure 7).

## Figures and Tables

**Table 1:** Primer sequences of target genes for mice.

Genes	Primer sequence (5′-3′)
*β-actin*	Forward: ACC GTG AAA AGA TGA CCC AGA
	Reverse: CCA GAG GCA TAC AGG GAC AA
*IL-1β*	Forward: ACT CAT TGT GGC TGT GGA GA
	Reverse: TTG TTC ATC TCG GAG CCT GT
*IL-6*	Forward: AGA CTT CCA TCC AGT TGC CT
	Reverse: CAG GTC TGT TGG GAG TGG TA
*TNF-α*	Forward: ACC CTC ACA CTC ACA AACCA
	Reverse: GGC AGA GAG GAG GTT GAC TT

**Table 2:** Concentration serum liver function biomarkers after KX treatment.

Groups	ALT (U/L)	AST (U/L)	ALB (g/L)	TP (g/L)
Control	24.58 ± 7.27	35.71 ± 5.66	42.58 ± 4.97	75.59 ± 9.04
Model	78.30 ± 11.06^##^	81.04 ± 15.02^##^	27.61 ± 7.03^#^	50.11 ± 10.37^##^
KX	45.41 ± 9.31^*∗∗*^	57.92 ± 9.88^*∗∗*^	36.52 ± 6.95^*∗*^	61.80 ± 9.80^*∗*^

Control, Model, and KX groups (*n* = 10 per group). Data are presented as mean ± SD. ^#^: *p* < 0.05 compared with the Control group; ^##^: *p* < 0.01 compared with the Control group; ^*∗*^: *p* < 0.05 compared with the Model group; ^*∗∗*^: *p* < 0.01 compared with the Model group.

**Table 3:** Concentration serum liver function biomarkers after antibiotics and KX treatment.

Groups	ALT (U/L)	AST (U/L)	ALB (g/L)	TP (g/L)
Control	28.51 ± 3.27	30.88 ± 9.83	38.18 ± 2.33	80.33 ± 9.58
Antibiotics	65.67 ± 15.66^##^	79.67 ± 18.71^##^	31.04 ± 5.06^#^	65.01 ± 15.99^#^
Antibiotics + KX	61.89 ± 18.55	71.85 ± 20.46	33.51 ± 3.75	59.48 ± 12.73

Control, Antibiotics, and Antibiotics + KX groups (*n* = 10 per group). Data are presented as mean ± SD. ^#^: *p* < 0.05 compared with the Control group; ^##^: *p* < 0.01 compared with the Control group.

## Data Availability

The authors declare that the datasets used and/or analyzed during the current study are available from the corresponding author on reasonable request.
